# Scirrhus of the Liver and Stomach, Tuberculosis, and Partial Fatty Degeneration of the Kidneys

**Published:** 1867

**Authors:** 


*Scirrhus of the Liver and Stomach, Tuberculosis, and Partial Fatty Degeneration of the Kidneys.* Hospital Record.

*History.*—John Manchester, aged 40 years, admitted Jan 23, 1866, states that some two or three months previous to admission, his health commenced failing, his appetite very much depraved, and he continually suffered more or less pain over the right hypochondriac region, and in the lower extremities. He has been continually getting worse up to the present time.

*Symptoms on Admission.*—Greatly emaciated, pale, anæmic, walks about with difficulty, articulates indistinctly, tremulous, muscles of the right side of the face relaxed, memory failing, questions must be repeated sometimes twice before he comprehends sufficiently to answer. Complains of pain in the lower extremities and abdomen, more especially over the epigastric and right hypochondriac regions. No appetite; great thirst; tongue coated with a yellowish fur; pulse 95 per minute, small and weak; respiration 16 per minute, somewhat labored. The physical signs revealed nothing abnormal about the chest; abnormal dullness over the right hypochondriac region. Evacuations from the bowels more or less frequent; the discharges fluid, and of a whitish color. Urinates frequently, but passes little at the time, causing slight pain. Does not vomit.

*Treatment.*—℞. Spt. frument. ℥ss, three times per diem, with pulvis doveri gr. vj. at night.

*Progress of the Case.—Jan. 24th.*—Slept well. Had two evacuations from the bowels during the night. Urinates often, but experiences less pain; traces of albumen in the urine. Gave the following:—℞. Tinct. cinch. comp, teaspoonful three times a-day, continuing spt. frument. and pulvis doveri at night.

*28th.*—Gradually growing worse; keeps the bed and sleeps most of the time. Articulation so indistinct that it is difficult to understand what he says. Indifferent as to taking nourishment.

*29th.*—Pulse 75; respiration 20 per minute, labored. ℞. Strych. puræ gr. ss., solv. in spt. frument. ℥ss., add aqua ℥j. S. Cne teaspoonful three times a-day.

*31st.*—Comatose, hard to rouse; respiration labored. ℞. Sherry wine ℥ss. every three hours. Discontinued previous treatment.

*Feb 1st.*—Died at 6 o'clock A.M.

*Sectio Cadaveris, 48 hours after Death.*—In the thorax, the heart was found very much contracted, especially the left ventricle. Lungs normal. In the abdominal cavity the liver was found converted throughout the greater portion of its extent into a scirrhous mass. Extensive scirrhus was also found throughout the entire curvature of the stomach. The upper third of the ascending and nearly the whole of the transverse colon were abnormally contracted. In both kidneys there was fatty degeneration. Bladder contracted and somewhat hypertrophied.

*History.*—Fred. Ronga, aged 31. Admitted Feb. 26, 1866. States that some eight weeks ago, while on shipboard, he was attacked with fever, which lasted for about three weeks. On the subsidence of the fever a severe cough ensued, which, in connection with pain over the chest and difficult breathing, has continued up to the present time.

*Symptoms on Admission.*—Greatly emaciated; anæmic; skin of a dusky hue; respiration hurried and exceedingly difficult, so he can rest only in a semi-sitting posture; pulse frequent, small, and weak; tongue coated with a whitish, yellow fur; slight dullness over the right lung, abnormal resonance over the left, throughout the whole of its extent; bowels somewhat irregular; urine scanty and highly colored; takes nourishment but sparingly; refuses to take stimulants; complains of pain over the abdomen; slight tympanites.

*Treatment.*—℞. Pulv. opii gr. $\frac{1}{2}$, quinia sulph. grs. iij., three times per diem, and milk-punch ℥j, every two hours.

*Progress of the Case.*—*Feb. 27.*—Did not sleep well during the night; complains of intense pain over the hypogastric region; no evacuations; catheter introduced into the bladder, which caused him some pain, flow of urine only limited; tongue dry, and symptoms generally indicated a low typhoid character. ℞. Emulsio terebinth. ℥j., three times per diem, contiuuing previous treatment.

*Feb. 28.*—Sinking very rapidly; refuses both nourishment and stimulants. Expression of the countenance anxious; respiration quick and labored. Expired at 4 o'clock A.M.

*Sectio Cadaveris, 24 hours after Death.*—Right lung slightly adherent to the chest anteriorly, left lung entirely collapsed, and on its anterior surface was an open cavity containing tuberculous matter, in the active stage of softening and suppurating. In the left pleural cavity, there was an oblong ovoid cyst, about 5 inches long and 3 inches in its transverse diameter, containing a serous, yellowish, translucent fluid. The membrane or capsule forming the cyst was of a yellow or buff color, its outer surface smooth, apparently having the character of a serous membrane; the inner surface rougher, and of a lighter color. The membrane was about $\frac{1}{16}$ of an inch thick, and so fragile that its own weight was sufficient to cause its rupture, under the attempt of removing it from the thoracic cavity. It had no vascular connections whatsoever, and was perfectly free from any attachment to the surrounding parts. Its location was above, to the left, and somewhat behind the heart, pressing the lung backwards and inwards. The glands of Peyer were dark-colored and congested, but no mark of ulceration. The liver gave evidence of commencing fatty degeneration. On the anterior surface of the right kidney, was an abscess half-an-inch in diameter, containing both pus and calcareous deposit. The line of demarcation between the cortical and medullary substance indistinct on both kidneys. The renal capsules easily separated.

*History.*—Kate Brown, aged 19, admitted February 5, 1866. States that about three years ago she contracted syphilis, from which she suffered for nearly four months, but finally recovered so well that she considered herself perfectly cured. About the 1st of July, last, her menses did not appear in proper time, and finally disappeared altogether. About the 1st of August, she commenced coughing, and suffered some pain over her chest; for a short time she raised blood. About the 1st of November, her throat became sore, so that she had difficulty in swallowing. Her general health, during all this time, continued to fail, and about the 1st of January, she had to keep her bed all the time. In the latter part of January, she lost her voice.

*Symptoms on Admission.*—Emaciated; hectic flush of the cheeks; coughs a good deal and raises a purulent, mucous sputa, but no blood; voice lost so she cannot speak above a loud whisper; great nervous irritability when speaking, and somewhat anxious expression of the face; bowels move five or six times during the day; tongue coated; mouth dry; lips sore; pulse frequent, 95, thready; respiration short; extensive ulceration of the larynx. On light percussion, there is abnormal resonance on the left side, but extensive dullness over both lungs on heavy percussion. Distinct cavernous respiration over the upper portion of both lungs.

*Treatment.*—Pulvis doveri grs. v., hydrarg. submuriat. gr. j., at night, followed by ol. ricin. ℥ss., in the morning.

*Progress of the Case.—Feb. 7.*—Diarrhœa continues. Ordered to take the following:—℞. Acid nitr. dilut. ʒj., tinct. opii ℥ss., syrup simpl. ℥iij. M.S. Teaspoonful 3 times a-day.

*Feb. 9.*—Diarrhœa considerably better. Complains of sore throat; the following gargle was ordered:—Potas. chl. ʒj., solve in aqua cam. ℥iv. S. Gargle the throat every three hours.

*Feb. 12.*—Diarrhœa entirely checked, ordered to take ol. morrh. and spirits frument., of each, ℥ss., three times per diem, discontinued previous treatment.

*March 1.*—Complains of piles, for which the following was ordered:—acid tannic. ʒj., morph. sulph. grs. x., unguent. hydrg. nitr. ʒj., adeps ℥ss. M. Ft. unguent. S. Apply twice a-day.

*March 10.*—Diarrhœa returned; cannot take the oil. ℞. Morph. sulph. grs. iij., acid sulph. aromat. ʒj., magnesia sulph. ʒij., syrup simpl. ℥j., aqua cam. ℥ss. M.S. Teaspoonful every four hours.

*March 12.*—Sinking very rapidly. Takes neither nourishment nor stimulants.

*March 13.*—Expired at 7 o'clock A.M.

*Sectio Cadaveris, 24 hours after Death.*—Greatly emaciated. In the thoracic cavity, the heart was normal. The lungs, with the exception of the lower margins, were one hard, solid, tuberculous mass, and, on section, were found to contain small sinuses filled with pus and calcareous deposits. In the abdomen, the stomach normal; intestines congested; no ulceration. Liver congested; tissue easily broken down. Commencing fatty degeneration of both kidneys, characterized by the usual structural changes, namely, obliteration of the line between the cortical and medullary portion, bloodlessness, color light, fawny, etc. In the larynx and trachea, there was extensive ulceration.

